# The High-Efficiency Degradation of Multiple Mycotoxins by Lac-W Laccase in the Presence of Mediators

**DOI:** 10.3390/toxins16110477

**Published:** 2024-11-04

**Authors:** Mengshuang Jia, Xiaohu Yu, Kun Xu, Xiaodan Gu, Nicholas J. Harmer, Youbao Zhao, Yuqiang Xiang, Xia Sheng, Chenglong Li, Xiang-Dang Du, Jiajia Pan, Wenbo Hao

**Affiliations:** 1 College of Veterinary Medicine, Henan Agricultural University, Zhengzhou 450046, China; 2 Living Systems Institute, University of Exeter, Exeter EX4 4QD, UK; 3 College of Sciences, Henan Agricultural University, Zhengzhou 450046, China

**Keywords:** laccase mediator system, detoxification, zearalenone

## Abstract

Mycotoxin cocontamination is a severe threat to health and economic security worldwide. The mycotoxins aflatoxin B_1_ (AFB_1_), zearalenone (ZEN), deoxynivalenol, T-2 toxin, fumonisin B_1_, and ochratoxin A are of particular concern due to their substantial toxicity. Lac-W is a laccase with the unique property of degrading these six mycotoxins in the absence of redox mediators. Nevertheless, their degradation rates are low. This work aims to improve the ability of Lac-W to degrade these six mycotoxins and to elucidate its detoxification mechanism. Including redox mediators increased the Lac-W degradation efficiency drastically, and completely degraded AFB_1_ and ZEN within one hour. Additionally, Lac-W-AS has good temperature, pH, and ions adaptability in ZEN degradation. Lac-W-AS reduced the ZEN toxicity because ZEN degradation products significantly restored the bioluminescence intensity of *Vibrio fischeri*. A Lac-W-AS-mediated oxidation product of ZEN was structurally characterized as 15-OH-ZEN by UHPLC-MS/MS. Linear sweep voltammetry showed that AS affected the potential of Lac-W and accelerated the oxidation of ZEN. Finally, the combination of mediators (acetosyringone and 2,2′-azino-bis (3-ethylbenzothiazoline-6-sulfonate)) improved the degradation rate of mycotoxins. This work highlights that the combination of Lac-W with mediators serves as a good candidate for degrading multi-mycotoxin contaminants in food and feedstuff.

## 1. Introduction

Mycotoxin cocontamination of agricultural commodities is a worldwide concern, owing to the serious health safety threat to livestock and humans and the substantial economic burden on animal husbandry and the food industry [1,2,3]. Multiple-mycotoxins cocontamination shows a comparatively high prevalence in cereal-based feedstuff and food [4]. Over a three-year period from 2018 to 2020, >75% of maize tested positive for multiple mycotoxin in different countries globally (Europe, Africa, Asia, and South America) [5]. Data from around 500,000 analyses from the European Food Safety Authority and an extensive global survey for multiple mycotoxin in cereals and nuts showed the occurrence of mycotoxins to be up to 60–80% [6]. Hundreds of types of mycotoxins with chemical heterogeneity have been identified [7]. However, not all of these mycotoxins are covered by legislation and guidelines issued in China, America, and the European Union. These jurisdictions focus on AFB_1_, ZEN, deoxynivalenol (DON), T-2 toxin, fumonisin B_1_ (FB_1_), and ochratoxin A (OTA) as these cause serious carcinogenic effects and organ damage, as well as their widespread prevalence [8,9]. Efficient mycotoxin detoxification strategies are therefore sought to secure global food supplies.

Strategies that have been applied to mitigate mycotoxin contamination include physical, chemical, and biological detoxification methods [10,11,12]. Microorganisms, in essence, microbial enzyme treatments, serve as a superior choice for mycotoxin detoxification. These have numerous advantages over conventional physical and chemical detoxification methods, including high efficiency, irreversibility, and lower environmental and physiological toxicity [13]. Many microbial enzymes have been reported that could degrade mycotoxins [14,15]. Most mycotoxin-degrading enzymes are specific to a particular type of mycotoxins, i.e., either AFB_1_, ZEN, or DON [16,17,18]. However, mycotoxin detoxification strategies must consider the co-occurrence of multiple mycotoxins. It is economically and environmentally superior to use one enzyme to degrade multiple mycotoxins. This is a challenge due to the diverse structures of mycotoxins. Many enzymes which can degrade multiple mycotoxins require cofactors or co-substrates. Dye-decolorizing peroxidase from *Bacillus subtilis* SCK6 degraded AFB_1_, ZEN, and DON when supplemented with Mn^2+^ [19]. Manganese peroxidases from fungi degraded AFB_1_, ZEN, DON, and FB_1_ in the dicarboxylic acid malonate [20]. Commercial peroxidase (*Armoracia rusticana*) degraded both ZEN and OTA when 26 mM H_2_O_2_ was added [21]. Oxidoreductases such as laccases are promiscuous in their substrates for oxidation and have the potential to simultaneously degrade multiple mycotoxins rather than only one type [22].

Laccases (EC 1.10.3.2) belong to the blue multicopper oxidases family and oxidate a variety of phenolic compounds, releasing water as a byproduct [23]. A few laccases have been reported to degrade one mycotoxin (AFB_1_) [16,24,25]. The range of laccase substrates can be further extended by constructing the laccase mediator system (LMS) using redox mediators, which are small molecule substrates for laccase [26,27]. The enhancement of the oxidation capacity of LMS might be due to the fact that the small molecular mediator first reacted with laccase to form free radicals, and then transferred electrons to the substrates, thereby reducing the steric hindrance of the substrates and enhancing the oxidation capacity of LMS [26]. Indeed, LMS has been used for mycotoxin detoxification [28,29,30]. It is reported that mediators can significantly accelerate the degradation of mycotoxins by laccase [19,31].

Our earlier study found that a laccase (Lac-W) from *Weizmannia coagulans* (*W. coagulans*) 36D1 directly degraded six major mycotoxins without mediators, namely AFB_1_, ZEN, DON, FB_1_, T-2 toxin, and OTA [32]. Compared to laccases and other microbial enzymes, Lac-W is highly cost-effective in production and robust for detoxifying mycotoxins. In this paper, we improved the degradation properties of Lac-W coupled with mediators to the above six mycotoxins, and optimized the conditions for ZEN degradation by LMS. Moreover, we elucidated the detoxification mechanism of ZEN by LMS, including the structure identification and toxicity analysis of ZEN degradation products, as well as the effect of the mediator on the Lac-W potential. We examined the effect of mediator combination and mycotoxin coexistence on the mycotoxin degradation by LMS. LMS was applied to feed-ingredient corn husk to explore the feasibility of LMS application in the food and feed industry. This work further demonstrates the exciting prospects for Lac-W in mycotoxin degradation, and shows that the development of Lac-W with improved properties could further raise their exploitation potential.

## 2. Results and Discussion

Our previous study showed that Lac-W degrades six mycotoxins with varied degradation rates, including AFB_1_ (88%), ZEN (60%), DON (34%), T-2 toxin (19%), FB_1_ (18%), and OTA (12%), after 24 h incubation without mediators [32]. When the reaction time was shortened to 1 h, the above six mycotoxins had almost no degradation. Given the mycotoxin degradation rates by Lac-W were not exceptionally high, different redox mediators were screened to assist in degrading mycotoxins in this study.

### 2.1. Mycotoxins Degradation by LMSs

#### 2.1.1. AFB_1_ Degradation by LMSs

**Figure 1:**
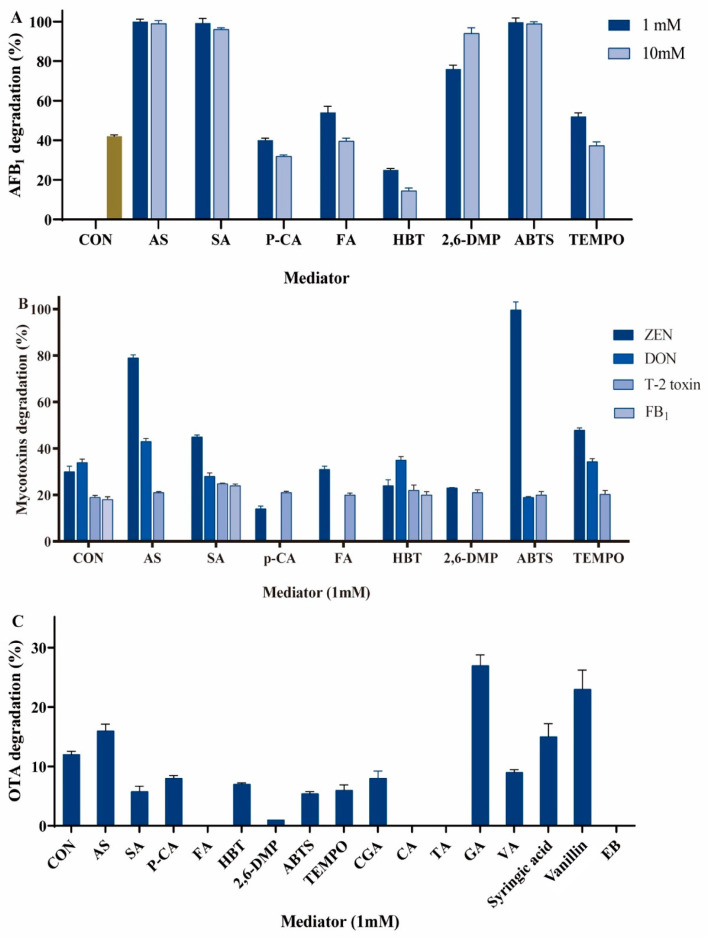
Mycotoxin degradations by LMSs. (**A**) AFB_1_ degradation by LMSs. (**B**) ZEN, DON, T-2 toxin, and FB_1_ degradation by LMSs. (**C**) OTA degradation by LMSs. Reaction condition: room temperature, initial mycotoxins 1 µg/mL, 1 U Lac-W and 1 mM or 10 mM mediator in 1 mL of Tris-HCl buffer (100 mM, pH 8.0) for 1 h at static condition. CON: controlled test, no mediator was added to the reaction system. Each reaction was repeated three times.

We tested eight redox mediators at concentrations of 1 mM or 10 mM in combination with Lac-W to test their ability in assisting Lac-W to degrade AFB_1_. AS (100%), SA (99%), 2, 6-DMP (95%), and ABTS (99%) were the best performing mediators, even when the reaction time was reduced to 1 h (Figure 1A). The AFB_1_ degradation rates were similar when the concentration of AS, SA, and ABTS were 1 mM or 10 mM (100% vs. 99% for AS, 99% vs. 97% for SA and 99% vs. 99% for ABTS). It was observed that 10 mM 2, 6-DMP has a higher degradation rate of AFB_1_ compared to 1 mM (95% for 10 mM vs. 76% for 1 mM). Therefore, 1 mM AS, SA and ABTS, and 10 mM 2, 6-DMP were applied for a cost-effective and efficient degradation. On the contrary, p-CA and HBT did not promote the AFB_1_ degradation but led to a decrease, compared with the AFB_1_ degradation by Lac-W in the absence of mediators. This may be related to the fact that efficient electron transport does not occur.

#### 2.1.2. ZEN Degradation by LMSs

According to the above results, 1 mM of ABTS, HBT, AS, SA, p-CA, FA, TEMPO, and 10 mM of 2, 6-DMP were assessed for their ability to assist Lac-W to degrade ZEN. In ABTS, the best mediator within the tested compounds, ZEN, was completely degraded (100%) within 1 h (Figure 1B). AS also effectively promoted the degradation of ZEN (79%). SA and TEMPO slightly improved the degradation of ZEN (45% for SA and 48% for TEMPO), whilst FA was ineffective, and p-CA, 2, 6-DMP, and HBT led to a decrease in ZEN degradation, compared with the ZEN degradation by Lac-W without a mediator.

#### 2.1.3. Degradation of DON, T-2 Toxin, FB_1_, and OTA by LMSs

The addition of 1 mM ABTS, AS, or HBT promoted the degradation of DON (Figure 1B). The degradation rates were 51% (ABTS), 43% (AS), 37% (TEMPO), and 35% (HBT), respectively, compared with the DON degradation by Lac-W (34%) without a mediator. In contrast, SA reduced the degradation rate of DON, and 2, 6-DMP, p-CA, and FA abolished DON degradation.

A slight improvement in the T-2 toxin degradation rate was observed with SA (25%), HBT (22%), AS (22%), 2, 6-DMP (21%), p-CA (21%), FA (20%), TEMPO (20%), and ABTS (20%), compared with no mediator (19%) (Figure 1B).

HBT and SA slightly increased the degradation rate of FB_1_. However, ABTS, AS, p-CA, FA, TEMPO, and 2, 6-DMP completely blocked the degradation of FB_1_ (Figure 1B).

OTA seemed to be the most recalcitrant mycotoxin to degradation (12%) by Lac-W. Of the eight redox mediators mentioned above, only AS was slightly effective as a mediator towards OTA degradation (16%) (Figure 1B). Accordingly, we added seven more mediators, including CGA, CA, TA, GA, vanilla alcohol, syringic acid, and vanillin. Among them, syringic acid (17%), vanillin (23%), and GA (27%) improved the OTA degradation rate, but the increase was still slight.

### 2.2. The Effects of Various Conditions on ZEN Degradation by Lac-W-AS

As expected, mediators strongly enhance the degrading ability of Lac-W on mycotoxins, especially AFB_1_ and ZEN. Lac-W-ABTS (99%), Lac-W-AS (100%), and Lac-W-SA (99%) were the most efficient among the eight mediator systems tested for AFB_1_ degradation. The Lac-W-ABTS and Lac-W-AS systems were the most efficient for ZEN degradation with a degradation rate of 99% and 79%, respectively. Given the Lac-W-SA system can efficiently degrade AFB_1_ and ZEN, and AS was a non-toxic natural phenol, we chose AS as a representative laccase mediator to investigate the performance of Lac-W to degrade ZEN, including culture mode, pH, temperature, concentration of Lac-W and AS, ions tolerance, and time process.

**Figure 2:**
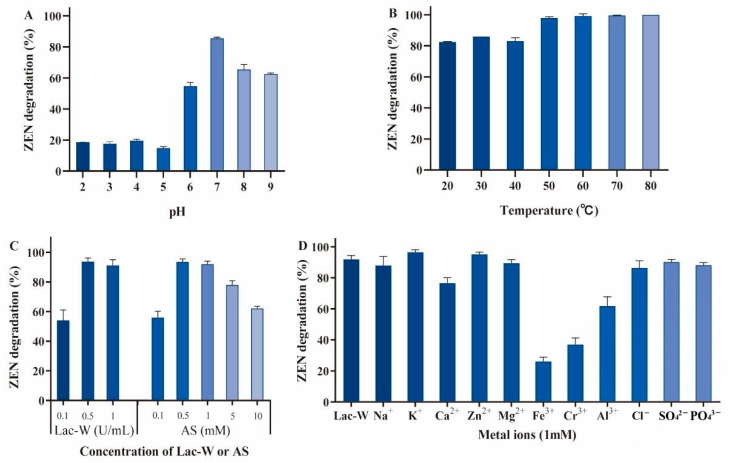
Effects of culture conditions on Lac-W-AS-mediated ZEN degradation. (**A**) Effect of pH on ZEN degradation. (**B**) Effect of temperature on ZEN degradation. (**C**) Effect of concentration of Lac-W and AS on ZEN degradation. (**D**) Effect of ions on ZEN degradation. The Lac-W was incubated with different ions at room temperature for 10 min, followed by ZEN. Reaction conditions: initial ZEN 1 µg/mL, culture conditions in static condition, pH 2.0–9.0, 20–80 °C, concentration of Lac-W or AS were 0.1–1 U/mL or 0.1–10 mM, and incubation time 1 h. The temperature, Lac-W/AS concentration, and metal ion assays were performed under optimal conditions derived from the pH, temperature, and Lac-W/AS reactions, respectively. Three independent replicates were performed for each assay. Each reaction was repeated three times.

Static conditions or 180 rpm did not affect the Lac-W-AS degradation of ZEN (Figure S1). pH had a great influence on the ZEN degradation. Lac-W-AS gave the highest degradation rate over a wide pH range of 6.0 to 9.0, and the optimal pH was 7.0 (Figure 2A). We also found that ZEN was unstable under strong alkaline conditions with pH > 9 (Figure S2). Lac-W-AS exhibited excellent temperature adaptability, and ZEN had a good degradation rate in the range of 20–80 °C (Figure 2B). We observed that 0.5 U/mL Lac-W was sufficient to degrade 1 µg/mL of ZEN, and 0.5 mM was the most appropriate AS addition amount (Figure 2C). Under the above optimal reaction conditions (static condition, pH 7.0, room temperature, 0.5 U/mL Lac-W, and 0.5 mM AS), 1 µg/mL ZEN was degraded 92% in 1 h. Furthermore, Lac-W-AS had good tolerance to both anions and cations, and some ions (K^+^ and Zn^2+^) promoted Lac-W-AS degradation of ZEN (Figure 2D).

### 2.3. Cytotoxicity Assay of ZEN Degradation Products

**Figure 3:**
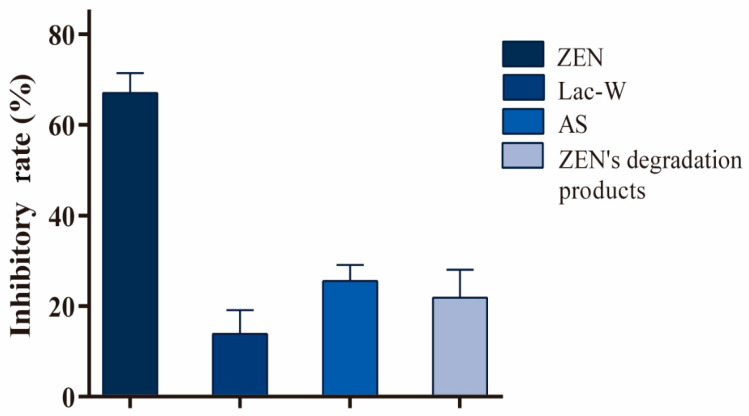
The inhibition rate of ZEN, Lac-W, AS, and ZEN’s degradation products against *Vibrio fischeri*. Each reaction was repeated six times.

To evaluate the cytotoxicity of ZEN degradation products by Lac-W-AS, and given that *Vibrio fischeri* has been reported to be useful for the evaluation of mycotoxin toxicity [15], we assessed the cytotoxicity of ZEN degradation products to *Vibrio fischeri*. The inhibition rate of ZEN degradation products against *Vibrio fischeri* was significantly lower than that of ZEN, and was comparable to that of the blank control, indicating that Lac-W-AS reduced the toxicity of ZEN (Figure 3). It has previously been reported that the laccase mediator system, composed of CotA laccase derived from *Bacillus subtilis* and methyl syringate, can detoxified ZEN [20].

### 2.4. ZEN Degradation Product by Lac-W-AS

**Figure 4:**
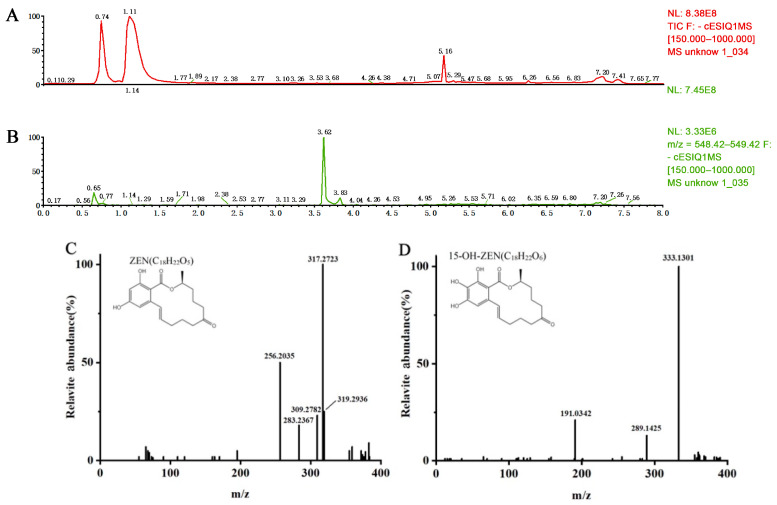
Mass spectra analysis of the ZEN degradation by Lac-W-AS. (**A**) Total ion chromatogram of ZEN (retention time of 5.16 min). (**B**) Total ion chromatogram of the ZEN degradation product (retention time of 3.62 min). (**C**) The mass spectra analysis of ZEN (C_18_H_22_O_5_; [M-H]^−^ with *m*/*z* 317.2715). (**D**) The mass spectra analysis of the ZEN degradation product by Lac-W (C_18_H_22_O_6_; [M-H]^−^ with *m*/*z* 333.1376). Mass spectrum parameters: ESI ion source, negative ion mode, and full scanning range 100–1500 *m*/*z*. The ion transfer tube temperature was set to 325 °C, and the vaporizer temperature was 350 °C. Orbitrap had a resolution of 120,000. The metabolite analysis software was Compound Discovery 3.0.

We elucidated the degradation products of ZEN by Lac-W-AS using high-resolution mass spectrometry. The retention time of ZEN was 5.16 min (Figure 4A), and its molecular ions *m*/*z* in mass spectrum was ([M-H]^−^) with 317.2723 (Figure 4C). An additional distinct peak at 3.62 min (Figure 4B) was found in the Lac-W-AS-mediated degradation of the ZEN sample, which was not detected in the reference ZEN. This peak revealed a molecular ion *m*/*z* of 333.1301 ([M-H]^−^) (Figure 4D), which was consistent with a molecular formula C_18_H_22_O_6_. This molecular formula indicated that hydroxylation was produced at position 13 or 15 of the aromatic ring of ZEN. However, 13-OH-ZEN was chemically unstable and auto-oxidizes to 13-OH-ZEN-quinone with a molecular ion *m*/*z* of 331. Thus, the degradation product of ZEN was presumed to be 15-OH-ZEN, and the daughter ions *m*/*z* of 289.1425 [M-44-H]^−^ and 191.0332 [M-142-H]^−^ were in accordance with the MS/MS fragments of 15-OH-ZEN [19].

### 2.5. Determination of the Redox Potential of Lac-W and Lac-W-AS

**Figure 5:**
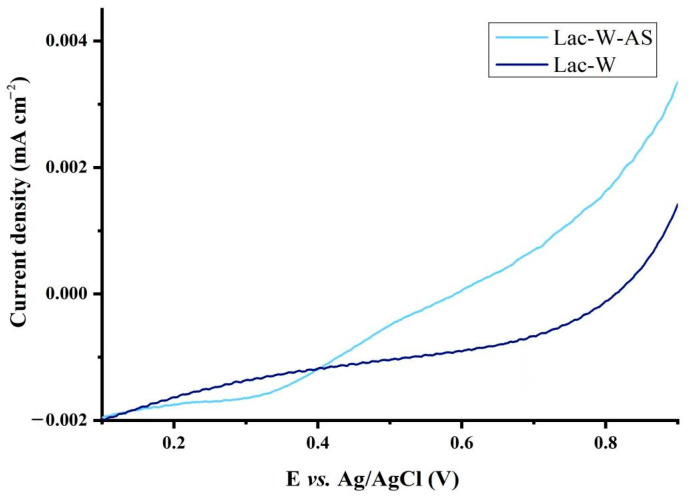
Determination of the redox potential of Lav-W and Lav-W-AS using linear sweep voltammetry.

The mediator efficacy in laccase-mediated oxidation is likely due to the effect of mediators on the redox potential of laccase, which can be determined by linear sweep voltammetry. The effects of AS on the experimental redox potential of laccase at pH 7.0 can be found in Figure 5. Lac-W-AS and ZEN started the oxidation reaction at 0.35 V, while Lac-W and ZEN started at 0.8 V, which indicated that Lac-W oxidation of ZEN is easier with AS addition.

### 2.6. Independent and Simultaneous Degradation of Mycotoxins by Lac-W-AS-ABTS

**Figure 6:**
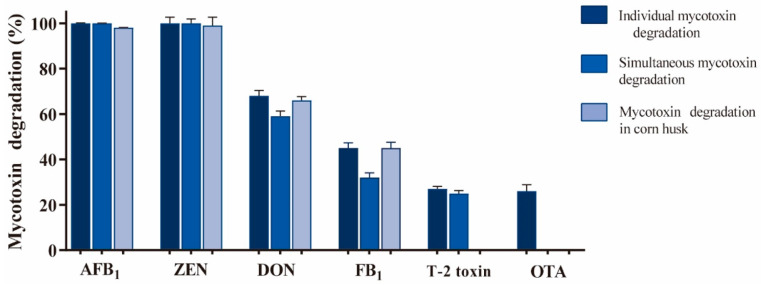
Mycotoxin degradation by Lac-W-AS-ABTS. Dark blue indicated individual mycotoxin degradation by Lac-W-AS-ABTS. Reaction conditions: room temperature, 1 µg/mL initial mycotoxins, 0.5 U Lac-W, 0.5 mM AS, and 0.5 mM ABTS in 1 mL of citrate–phosphate buffer (100 mM, pH 7.0) for 1 h at static condition. Medium blue indicated simultaneous mycotoxin degradation by Lac-W-AS-ABTS. Reaction conditions: 1 µg/mL each of initial mycotoxins, 3 U Lac-W, 3 mM AS, and 3 mM ABTS in 6 mL of buffer. Light blue indicated mycotoxin degradation in corn husk by Lac-W-AS-ABTS. Reaction conditions: 2 g corn husk, 2 U Lac-W, 2 mM AS, and 2 mM ABTS in 50 mL of buffer. Each reaction was repeated three times.

Given that AS and ABTS were the best mediators for the above mycotoxin degradation, and given that they were two types of mediator (a natural phenol deriving from plants and an artificial compound), their combination might have a better effect on mycotoxin degradation. We attempted to further improve the degradation rate of the six mycotoxins tested by Lac-W with AS and ABTS combined as redox mediators. The results showed that the degradation rates of mycotoxins were 100% for AFB_1_, 100% for ZEN, 68% for DON, 45% for FB_1_, 27% for T-2 toxin, and 26% for OTA (Figure 6). Compared with the single mediator AS or ABTS, the combination of AS and ABTS increased the degradation rates of DON (from 51% to 68%), FB_1_ (from 24% to 45%), T-2 toxin (from 22% to 27%), and OTA (from 16% to 26%). At the same time, AFB_1_ and ZEN were not affected and were still completely degraded. There might be additive or synergistic effects when two mediators were present in a system. Thus, combining different mediators was an active strategy to improve mycotoxin degradation.

The simultaneous degradation of these six mycotoxins by Lac-W-AS-ABTS in one system was determined. The results showed that AFB_1_ and ZEN were unaffected and degraded completely, and other mycotoxin degradation rates decreased slightly (Figure 6). The degradation rates were reduced from 68% to 59% for DON, from 45% to 32% for FB_1_, from 27% to 25% for T-2 toxin, and from 26% to 0% for OTA compared to the single degradation assay. Similar findings have been reported earlier [33]. This might be due to the fact that the six mycotoxins were all substrates of Lac-W and interfere with each other when reacting with Lac-W-AS-ABTS.

### 2.7. Mycotoxin Degradation in Corn Husk by Lac-W-AS-ABTS

Under the optimized mediators’ combinations conditions, Lac-W-AS-ABTS was used to degrade mycotoxins in contaminated corn husk. A total of 41 ng/g AFB_1_, 948 ng/g ZEN, 940 ng/g DON, 55 ng/g FB_1_, and no T-2 toxin and OTA were detected in the corn husks. After reacting with Lac-W-AS-ABTS for 1 h, the corn husk contained 1 ng/g AFB_1_, 14 ng/g ZEN, 321 ng/g DON, and 30 ng/g FB_1_. The degradation capacity of AFB_1_ was 40 ng/g, that of ZEN was 934 ng/g, that of DON was 619 ng/g, and that of FB_1_ was 25 ng/g, and the degradation rates were 98% for AFB_1_, 99% for ZEN, 66% for DON, and 45% for FB_1_, respectively (Figure 6). This was consistent with the degradation of mycotoxin standards in the buffer medium.

## 3. Discussion

It has been reported that mediators can promote the efficient degradation of AFB_1_ and ZEN by laccase. An amount of 1 μg/mL AFB_1_ was degraded by Ery4 laccase from *Saccharomyces cerevisiae* ITEM 17289 using 10 mM AS (73%) and SA (68%) as redox mediators; however, the Ery4 could not degrade AFB_1_ without a mediator [33]. Subsequently, 100% of degradation for 0.1 μg/mL AFB_1_ was obtained by Loi using Ery 4 laccase with 10 mM AS as mediator [29] (Loi et al., 2023). AFB_1_ was degraded by Lac2 from *Pleurotus pulmonarius* with 10 mM AS (90%), SA (72%), or ABTS (81%), and ABTS or AS assisted in the degradation of 100% or 88% ZEN [28,34]. Wang et al. showed that methyl syringate assisted *Bs*CotA to degrade 98% of AFB_1_ and 100% of ZEN [30].

Loi et al. attempted to degrade DON using laccase combined with various mediators with different mechanisms without success [33]. Sun et al. used molecular docking simulations to suggest that laccase from *Triticum aestivum* interacted with DON, and identified it as a trapper for DON, but this hypothesis had yet to be proved experimentally [35]. Shanakhat et al. reported that TEMPO was the most effective mediator for fungal laccases degradation of DON, degrading 60% of DON [31]. However, in this study, TEMPO only slightly promoted the degradation of DON by Lac-W. This might be attributed to the differences in the source and redox potential of laccases. Lac-W was derived from bacteria, while the laccase in Shanakhat’s paper was derived from fungi. The weak oxidation of laccase and LMS makes it difficult for DON to be oxidized. The oxidizing capacity of oxidoreductases was higher than that of laccase and LMS, such as peroxidase of *Aspergillus oryzae* and *Rhizopusoryzae,* which were positively correlated with DON degradation [36].

Decontamination strategies for T-2 toxins include physical adsorption, chemical, and biological techniques [37]. However, so far, there have been no other reports of enzyme biotransformation of T-2 toxin, except by Loi et al. who reported that T-2 toxin was slightly degraded by Ery4 laccase with TEMPO (40%) and ABTS (13%) [33].

Loi et al. reported that Ery4 laccase containing 1 mM SA degraded FB_1_ (25%) [33], which was similar to our experimental results (24% for Lac-W with SA). Heinl et al. reported that FB_1_ was degraded by the consecutive action of de-esterification and deamination with carboxylesterase from *Sphingopyxis* sp. MTA144 [38].

Since we had yet to find a suitable mediator for Lac-W, Lac-W was not an effective enzyme for OTA degradation. Cho et al. reported that crude *Aspergillus tubingensis* M036 and M074 enzymes removed 97.5% and 91.3% OTA (0.04 µg/mL) within 24 h, respectively [39]. The most critical OTA biodegradation mechanism is the degradation of OTA to non-toxic or less toxic OTα via the hydrolysis of the amide bond [40].

In our previous study, we found that the substrate binding domain of Lac-W was large enough to allow ZEN to enter, but the affinity between Lac-W and ZEN was weaker than that between Lac-W and AFB_1_, so the degradation efficiency of AFB_1_ by Lac-W was higher than that of ZEN [32]. AS is a low molecular weight organic molecule, which increases its ability to rapidly reach Lac-W’s active site. We speculated that AS accelerated the oxidation rate of Lac-W to ZEN through its electron transfer. The redox potential reflected oxidation capacity and strength; the higher the redox potential, the stronger the oxidation. High redox potential laccases had stronger oxidation capacity and a wider substrate spectrum, and oxidized more substrates [41]. The mediator can also enlarge the substrate spectrum of laccases by increasing the redox potential. For example, laccase (0.5–0.8 V) first oxidized ABTS (0.472 V) to ABTS^+^· and then into ABTS^++^ (0.855 V), which has a high redox potential and oxidized more substrates [42]. Laccase first oxidized AS (0.534 V) to aryloxy radicals, then to 2, 6-dimethoxy-*p*-hydroquinone, and finally into 2, 6-dimethoxy-*p*-benzoquinone (2, 6-DMBQ), which further oxidized the substrates [43]. Both AS and ABTS are oxidized to radicals by laccase first, but the oxidation capacity of AS to ZEN is weaker than that of ABTS, which may be associated with the lower redox potential of 2, 6-DMBQ than ABTS^++^.

## 4. Conclusions

This study demonstrated that redox mediators improved the catalytic efficiency of Lac-W significantly, and degraded AFB_1_ and ZEN completely within 1 h. Lac-W-AS has good temperature, pH, and ions adaptability in ZEN degradation, and reduced the toxicity of ZEN. A Lac-W-AS-mediated oxidation product of ZEN was structurally characterized as 15-OH-ZEN. AS affected the potential of Lac-W and accelerated the oxidation of ZEN. The AS and ABTS combination improved the degradation rate of mycotoxins. Where mycotoxins coexist, the degradation rates were slightly reduced. The same effect was observed in corn husk. This study provided a way to improve the degradation performance of laccases, making it better counteract multiple mycotoxins in food and feed.

## 5. Materials and Methods

### 5.1. Chemicals, Recombined Bacteria Strain, and Mycotoxins Standard

The 2,2′-azino-bis (3-ethylbenzothiazoline-6-sulfonate) (ABTS) was purchased from Macklin (Shanghai, China). The 2,6-dimethoxyphenol (2,6-DMP) was purchased from Sigma-Aldrich (Shanghai, China). Vanilla alcohol (VA), p-coumaric acid (p-CA), 1-hydroxybenzotriazole (HBT), acetosyringone (AS), syringaldehyde (SA), chlorogenic acid (CGA), ferulic acid (FA), caffeic acid (CA), syringic acid, tannic acid (TA), TEMPO, vanillin, and gallic acid (GA) were purchased from Solarbio (Beijing, China). Standards of AFB_1_, ZEN, DON, OTA, FB_1_, and T-2 toxin were all purchased from Pribolab (Qingdao, China). Lac-W was expressed, purified, and stored in our laboratory as previously described [32]. *Vibrio fischeri* CICC 10483 was purchased from the China Center of Industrial Culture Collection (CCICC, Beijing, China). Methanol and acetonitrile (UPLC grade) and all other analytical grade or higher reagents were purchased from Sinopharm (Shanghai, China).

### 5.2. Mycotoxins Degradation by LMS

Eight LMS were constructed by combining 1 U/mL Lac-W with 1 mM of four natural mediators (AS, SA, p-CA, and FA) or four artificial ones (ABTS, HBT, TEMPO, and 2,6-DMP), respectively. Their ability to degrade AFB_1_, ZEN, DON, FB_1_, and T-2 toxin was tested using an in vitro assay. OTA degradation was assayed with fifteen mediators, including the above eight intermediates, as well as CGA, CA, TA, GA, VA, syringic acid, and vanillin. A total of 1 µg/mL AFB_1_, ZEN, DON, OTA, FB_1_, or T-2 toxin, combined with 1 U/mL of Lac-W and 1 mM mediator were added in 1 mL 100 mM Tris-HCl buffer (pH 8.0). One unit of Lac-W activity is defined as the amount of Lac-W required to oxidize 1 µmol ABTS per minute. The reaction conditions were 1 h static condition at room temperature. The reaction was terminated by adding three volumes of methanol to the reaction. The controlled trial was an equivalent amount of heat inactivated Lac-W instead of Lac-W.

The effects of various conditions on ZEN degradation by Lac-W with AS were studied, including culture conditions (static or 180 rpm), pH (glycine-HCl for pH 2.0, pH citrate–phosphate for 3.0–7.0, Tris-HCl for pH 8.0–9.0, glycine-NaOH for pH 10.0–12.0), temperature (20–80 °C), concentration of Lac-W (0.1, 0.5, 1 U/mL), concentration of AS (1, 5, 10 mM), and metal ions or anions (1 mM).

The simultaneous degradation of this six mycotoxins combination was assessed using AS and ABTS in one system. A total of 1 µg/mL AFB_1_, ZEN, DON, OTA, FB_1_, and T-2 toxin, combined with 6 U/mL of Lac-W, 6 mM AS, and 6 mM ABTS, were added in 6 mL 100 mM Tris-HCl buffer (pH 8.0).

### 5.3. Mycotoxin Degradation in Corn Husk

Under the optimized mediators’ combination condition, LMS was used to degrade multiple mycotoxins in contaminated corn husk. An amount of 2 g corn husks were ground into powder, and 50 mL pH 7.0 citrate–phosphate buffer, 2 U Lac-W, 2 mM AS, and 2 mM ABTS were added to it. The room temperature was static 1 h. After filtration with ordinary filter paper and glass fiber filter paper, the filtrate was enriched and purified by immunoaffinity columns of different mycotoxins. After the filtrate was dried with nitrogen at 50 °C, 1 mL of 10% (*v*/*v*) acetonitrile solution was added and filtered to 0.22 μm, following which UHPLC-MS/MS was used for detection. The control reactions were thermal inactivated Lac-W.

### 5.4. Evaluation of Mycotoxin Degradation Rates by UHPLC-MS/MS

The mycotoxin concentrations in the LMS-mediated degradation reactions were determined by an ultra-high-performance liquid chromatography (ACQUITY UHPLC System, Waters Corp., Milford, CT, USA) coupled with a three-stage quadrupole mass spectrometer (I-CLASS PLUS/XEVO TQ-XS, Waters Corp., Milford, CT, USA). The test was repeated three times for each sample. The UHPLC system had an ACQUITY UPLC^®^BEH C_18_ column (1.7 μm, 2.1 × 50 mm) (Waters Corp., Milford, CT, USA). ESI-MS positive ionization mode was performed for AFB_1_, DON, FB_1_, T-2 toxin, and the negative ionization mode for ZEN and OTA. The samples were filtered by an RC 0.22 μm filter, and 5 μL of volume was injected. A flow rate of 0.3 mL/min was used. The mobile phase of AFB_1_, DON, FB_1_, and T-2 toxin consisted of acetonitrile and ultrapure water containing 0.1% (*v*/*v*) formic acid. The mobile phase of ZEN and OTA consisted of acetonitrile and ultrapure water. The following gradient was applied: 1–3 min from 10 to 90% (*v*/*v*) acetonitrile, 3–5 min 90% (*v*/*v*) acetonitrile, and 5–7 min from 90 to 10% (*v*/*v*) acetonitrile. The formula of the mycotoxin degradation rate was D = (1 − D_e_/D_c_) × 100%. D was the degradation rate, and D_e_ and D_c_ were the mycotoxin peaks in the experimental group and control group, respectively.

### 5.5. Luminescence Inhibition Assays

The luminescent bacterium *Vibrio fischeri* (CICC 10483) was used to evaluate the cell toxicity of the degradation products of ZEN. An amount of 1 mL of *Vibrio fischeri* was mixed with 300 µg/mL ZEN, 75 U Lac-W, 75 mM AS, or 300 µg/mL ZEN biotransformation products by Lac-W-AS, and incubated for 4 h in a 5 mL tube. A Varioskan LUX Multimode Microplate Reader (Thermo Fisher Scientific, Waltham, MA, USA) was used to measure the luminosity of each sample, with three technical repeats. The blank control was 0.8% (*w*/*v*) NaCl. The formula of luminescence inhibition percentage was I = (1 − I_e_/I_c_) × 100%. I was the luminescence inhibition ratio, and I_e_ and I_c_ were the luminescence intensities of sample and control, respectively.

### 5.6. Evaluation of ZEN Degradation Product by LMS

The ZEN degradation product was analyzed by UHPLC-MS/MS (Q Exactive Plus Orbitrap System, Thermo Scientific^TM^), performed in negative ion mode. ZEN and its degradation product were resolved on an ACQUITY UPLC^®^BEH C_18_ column (1.7 μm, 2.1 × 50 mm). A ZEN degradation product sample containing 1 μg/mL ZEN, 0.5 mM AS, and 0.5 U/mL Lac-W in 1 mL ultrapure water (pH 7.0) was incubated at room temperature for 8 h. Samples were filtered to 0.22 μm and 5 μL was injected into the system. The UHPLC separation conditions were the same as in Section 2.4. A full-scan mass spectrum was obtained in the range of 100–1500 *m*/*z*. The ion transfer tube temperature was set to 325 °C, and the vaporizer temperature was 350 °C. Orbitrap had a resolution of 120,000. The metabolite analysis software was Compound Discovery 3.0.

### 5.7. Linear Sweep Voltammetry

Linear sweep voltammetry (LSV) experiments were carried out with a CHI760E electrochemical workstation (CH Instruments, Shanghai, China), with a working electrode with glass carbon electrode supported by catalyst, an Ag/AgCl reference electrode, and a platinum counter electrode (wire, Ø0.5 mm). The scan rate was 50 mV/s from 0–0.9 V in 10 mM HEPES buffer at pH 7.0.

## Data Availability

The original contributions presented in this study are included in the article/Supplementary Material. Further inquiries can be directed to the corresponding authors.

## Supplementary Materials

The following supporting information can be downloaded at: https://www.mdpi.com/article/10.3390/toxins16110477/s1, Figure S1: effect of culture conditions on ZEN degradation by Lac-W-AS; Figure S2: the ZEN stability against pH.

## References

[1] Li J., Deng Y., Wang Y., Nepovimova E., Wu Q., Kuca K. (2023). Mycotoxins have a potential of inducing cell senescence: A new understanding of mycotoxin immunotoxicity. Environ. Toxicol. Pharmacol..

[2] Stuhldreier F., Schmitt L., Lenz T., Hinxlage I., Zimmermann M., Wollnitzke P., Schliehe-Diecks J., Liu Y., Jäger P., Geyh S. (2022). The mycotoxin viriditoxin induces leukemia- and lymphoma-specific apoptosis by targeting mitochondrial metabolism. Cell Death Dis..

[3] Johns L.E., Bebber D.P., Gurr S.J., Brown N.A. (2022). Emerging health threat and cost of Fusarium mycotoxins in European wheat. Nat. Food.

[4] Yang C., Song G., Lim W. (2020). Effects of mycotoxin-contaminated feed on farm animals. J. Hazard. Mater..

[5] Raj J., Farkaš H., Jakovčević Z., Medina A., Magan N., Čepela R., Vasiljević M. (2022). Comparison of multiple mycotoxins in harvested maize samples in three years (2018–2020) in four continents. Food Addit. Contam. Part A.

[6] Eskola M., Kos G., Elliott C.T., Hajšlová J., Mayar S., Krska R. (2020). Worldwide contamination of food-crops with mycotoxins: Validity of the widely cited ‘FAO estimate’ of 25. Crit. Rev. Food Sci. Nutr..

[7] Juraschek L.M., Kappenberg A., Amelung W. (2022). Mycotoxins in soil and environment. Sci. Total Environ..

[8] Feng C., Bai H., Chang X., Wu Z., Dong W., Ma Q., Yang J. (2023). Aflatoxin B1-induced early developmental hepatotoxicity in larvae zebrafish. Chemosphere.

[9] Jia B., Lin H., Yu S., Liu N., Yu D., Wu A. (2023). Mycotoxin deoxynivalenol-induced intestinal flora disorders, dysfunction and organ damage in broilers and pigs. J. Hazard Mater..

[10] Liu L., Xie M., Wei D. (2022). Biological Detoxification of Mycotoxins: Current Status and Future Advances. Int. J. Mol. Sci..

[11] Mao J., Zhou Y., Lv G., Zhou R. (2022). Simultaneous Detoxification of Aflatoxin B1, Zearalenone and Deoxynivalenol by Modified Montmorillonites. Molecules.

[12] Yu Y., Shi J., Xie B., He Y., Qin Y., Wang D., Shi H., Ke Y., Sun Q. (2020). Detoxification of aflatoxin B1 in corn by chlorine dioxide gas. Food Chem..

[13] Sánchez-Arroyo A., Plaza-Vinuesa L., Rivas B.L., Mancheño J.M., Muñoz R. (2023). The salicylate 1,2-dioxygenase from Pseudaminobacter salicylatoxidans DSM 6986T is a bifunctional enzyme that inactivates the mycotoxin ochratoxin A by a novel amidohydrolase activity. Int. J. Biol. Macromol..

[14] Shi Y., Ouyang B., Zhang Y., Zhang W., Xu W., Mu W. (2024). Recent developments of mycotoxin-degrading enzymes: Identification, preparation and application. Crit. Rev. Food Sci. Nutr..

[15] Adegoke T.V., Yang B., Tian X., Yang S., Gao Y., Ma J., Wang G., Si P., Li R., Xing F. (2023). Simultaneous degradation of aflatoxin B1 and zearalenone by Porin and Peroxiredoxin enzymes cloned from Acinetobacter nosocomialis Y1. J. Hazard. Mater..

[16] Zaccaria M., Dawson W., Russel Kish D., Reverberi M., Bonaccorsi di Patti M.C., Domin M., Cristiglio V., Chan B., Dellafiora L., Gabel F. (2023). Experimental-theoretical study of laccase as a detoxifier of aflatoxins. Sci. Rep..

[17] Fang Y., Zhang Z., Xu W., Zhang W., Guang C., Mu W. (2022). Zearalenone lactonase: Characteristics, modification, and application. Appl. Microbiol. Biotechnol..

[18] Li D., Liang G., Mu P., Lin J., Huang J., Guo C., Li Y., Lin R., Jiang J., Wu J. (2023). Improvement of catalytic activity of sorbose dehydrogenase for deoxynivalenol degradation by rational design. Food Chem..

[19] Qin X., Xin Y., Su X., Wang X., Wang Y., Zhang J., Tu T., Yao B., Luo H., Huang H. (2021). Efficient Degradation of Zearalenone by Dye-Decolorizing Peroxidase from Streptomyces thermocarboxydus Combining Catalytic Properties of Manganese Peroxidase and Laccase. Toxins.

[20] Wang X., Qin X., Hao Z., Luo H., Yao B., Su X. (2019). Degradation of Four Major Mycotoxins by Eight Manganese Peroxidases in Presence of a Dicarboxylic Acid. Toxins.

[21] de Oliveira Garcia S., Sibaja K.V.M., Nogueira W.V., Feltrin A.C.P., Pinheiro D.F.A., Cerqueira M.B.R., Badiale Furlong E., Garda-Buffon J. (2020). Peroxidase as a simultaneous degradation agent of ochratoxin A and zearalenone applied to model solution and beer. Food Res. Int..

[22] Ren D., Wang Z., Jiang S., Yu H., Zhang S., Zhang X. (2020). Recent environmental applications of and development prospects for immobilized laccase: A review. Biotechnol. Genet Eng. Rev..

[23] Dong C.D., Tiwari A., Anisha G.S., Chen C.W., Singh A., Haldar D., Patel A.K., Singhania R.R. (2023). Laccase: A potential biocatalyst for pollutant degradation. Environ. Pollut..

[24] Xiong D., Wen J., Lu G., Li T., Long M. (2022). Isolation, Purification, and Characterization of a Laccase-Degrading Aflatoxin B1 from Bacillus amyloliquefaciens B10. Toxins.

[25] Bian L., Zheng M., Chang T., Zhou J., Zhang C. (2022). Degradation of Aflatoxin B1 by recombinant laccase extracellular produced from *Escherichia coli*. Ecotoxicol. Environ. Saf..

[26] Wang X., Meng F., Zhang B., Xia Y. (2023). Elimination of tetracyclines in seawater by laccase-mediator system. Chemosphere.

[27] Gigli V., Piccinino D., Avitabile D., Antiochia R., Capecchi E., Saladino R. (2022). Laccase Mediator Cocktail System as a Sustainable Skin Whitening Agent for Deep Eumelanin Decolorization. Int. J. Mol. Sci..

[28] Song Y., Wang Y., Guo Y., Qiao Y., Ma Q., Ji C., Zhao L. (2021). Degradation of zearalenone and aflatoxin B1 by Lac2 from Pleurotus pulmonarius in the presence of mediators. Toxicon.

[29] Loi M., De Leonardis S., Ciasca B., Paciolla C., Mulè G., Haidukowski M. (2023). Aflatoxin B1 Degradation by Ery4 Laccase: From In Vitro to Contaminated Corn. Toxins.

[30] Wang X., Bai Y., Huang H., Tu T., Wang Y., Wang Y., Luo H., Yao B., Su X. (2019). Degradation of Aflatoxin B1 and Zearalenone by Bacterial and Fungal Laccases in Presence of Structurally Defined Chemicals and Complex Natural Mediators. Toxins.

[31] Shanakhat H., McCormick S.P., Busman M., Rich J.O., Bakker M.G. (2022). Modification of Deoxynivalenol by a Fungal Laccase Paired with Redox Mediator TEMPO. Toxins.

[32] Hao W.B., Gu X., Yu X., Zhao Y., Li C., Jia M., Du X.D. (2023). Laccase Lac-W detoxifies aflatoxin B1 and degrades five other major mycotoxins in the absence of redox mediators. Environ. Pollut..

[33] Loi M., Quintieri L., Fanelli F., Caputo L., Mulè G. (2018). Application of a recombinant laccase-chlorogenic acid system in protein crosslink and antioxidant properties of the curd. Food Res. Int..

[34] Loi M., Fanelli F., Zucca P., Liuzzi V.C., Quintieri L., Cimmarusti M.T., Monaci L., Haidukowski M., Logrieco A.F., Sanjust E. (2016). Aflatoxin B1 and M1 Degradation by Lac2 from Pleurotus pulmonarius and Redox Mediators. Toxins.

[35] Sun Z., Zhou Y., Hu Y., Jiang N., Hu S., Li L., Li T. (2022). Identification of Wheat LACCASEs in Response to Fusarium graminearum as Potential Deoxynivalenol Trappers. Front. Plant Sci..

[36] Garda-Buffon J., Kupski L., Adiale-Furlong E. (2011). Deoxynivalenol (don) degradation and peroxidase enzyme activity in submerged fermentation. Food Sci. Technol. Int..

[37] Janik E., Niemcewicz M., Podogrocki M., Ceremuga M., Stela M., Bijak M. (2021). T-2 Toxin-The Most Toxic Trichothecene Mycotoxin: Metabolism, Toxicity, and Decontamination Strategies. Molecules.

[38] Heinl S., Hartinger D., Thamhesl M., Vekiru E., Krska R., Schatzmayr G., Moll W.D., Grabherr R. (2010). Degradation of fumonisin B1 by the consecutive action of two bacterial enzymes. J. Biotechnol..

[39] Cho S.M., Jeong S.E., Lee K.R., Sudhani H.P., Kim M., Hong S.Y., Chung S.H. (2016). Biodegradation of Ochratoxin A by Aspergillus tubingensis Isolated from Meju. J. Microbiol. Biotechnol..

[40] Chen W., Li C., Zhang B., Zhou Z., Shen Y., Liao X., Yang J., Wang Y., Li X., Li Y. (2018). Advances in Biodetoxification of Ochratoxin A-A Review of the Past Five Decades. Front. Microbiol..

[41] Guebitz G.M. (2021). Oxidation of various kraft lignins with a bacterial laccase enzyme. Int. J. Mol. Sci..

[42] Fabbrini M., Galli C., Gentili P. (2002). Comparing the catalytic efficiency of some mediators of laccase. J. Mol. Catal. B Enzym..

[43] Pezzella C., Guarino L., Piscitelli A. (2015). How to enjoy laccases. Cell Mol. Life Sci..

