# Supplementary material for: The High-Efficiency Degradation of Multiple Mycotoxins by Lac-W Laccase in the Presence of Mediators
Source: Toxins (Basel). 2024 Nov 4;16(11):477. doi: 10.3390/toxins16110477 (PMC11598361; doi:10.3390/toxins16110477)
Supplement: Supplementary file 1 [file toxins-16-00477-s001.zip › toxins-3272023-supplementary.pdf]

# Supplementary Materials: The High-Efficiency Degradation of Multiple Mycotoxins by Lac-W Laccase in the Presence of Mediators

**Mengshuang Jia, Xiaohu Yu, Kun Xu, Xiaodan Gu, Nicholas J Harmer, Youbao Zhao, Yuqiang Xiang, Xia Sheng, Chenglong Li, Xiang-Dang Du, Jiajia Pan and Wenbo Hao**

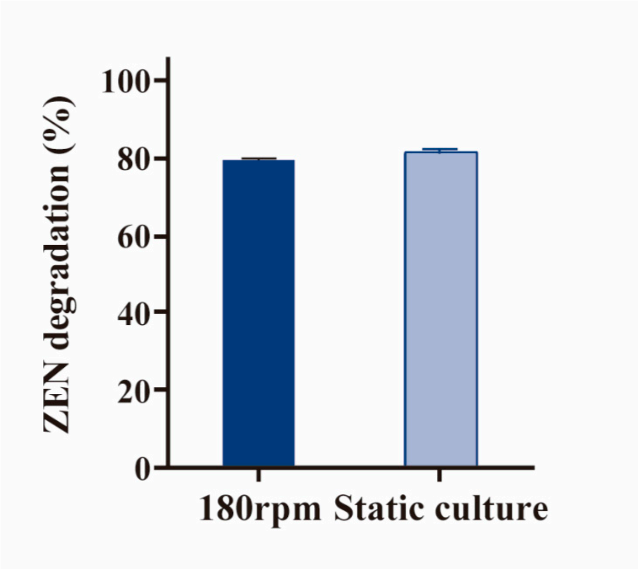


**Figure S1.** Effect of culture conditions on ZEN degradation by Lac-W-AS.

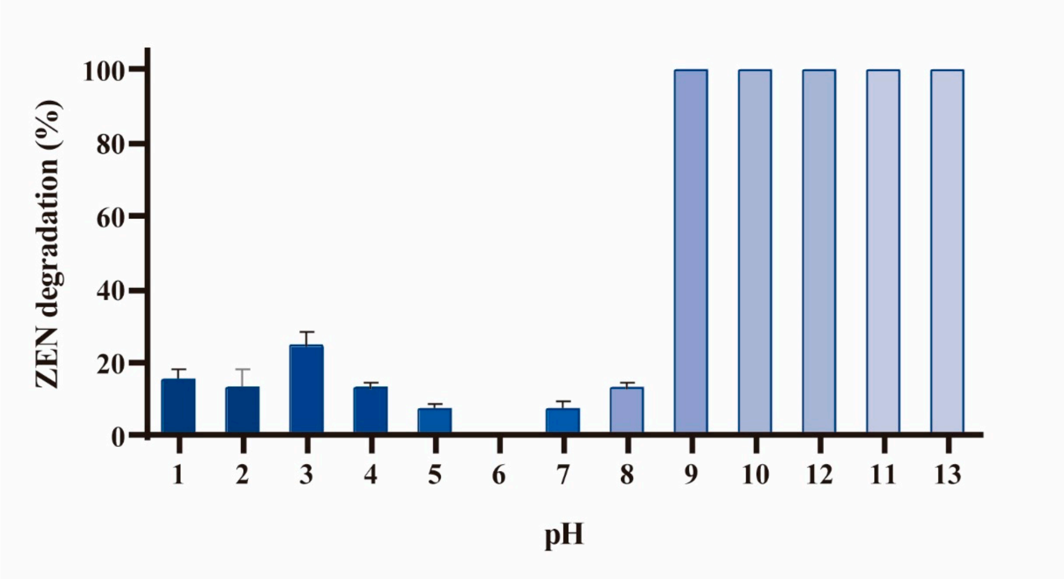


**Figure S2.** The ZEN stability against pH. 1 μg/mL ZEN was cultured with different pH at room temperature and 180 rpm for 4 h.
